# Highly sensitive hydrogen analysis employing low pressure laser induced breakdown spectroscopy in helium surrounding gas under electric field

**DOI:** 10.1038/s41598-025-11419-7

**Published:** 2025-07-22

**Authors:** Indra Karnadi, Marincan Pardede, Rinda Hedwig, Ivan Tanra, Maria Margaretha Suliyanti, Marvin Yonathan Hadiyanto, Eric Jobiliong, Ihan Martoyo, Zener Sukra Lie, Kiichiro Kagawa, Tjung Jie Lie, Koo Hendrik Kurniawan

**Affiliations:** 1 Department of Electrical Engineering, Krida Wacana Christian University, Jakarta, 11470 Indonesia; 2 Department of Electrical Engineering, University of Pelita Harapan, 1100 M.H. Thamrin Boulevard, Lippo Village, Tangerang, 15811 Indonesia; 3 Computer Engineering Department, Faculty of Engineering, Bina Nusantara University, Jakarta, 14810 Indonesia; 4 Research Center for Photonics – National Research and Innovation Agency, KST BJ Habibie, South Tangerang, 15314 Indonesia; 5 Automotive and Robotic Program, Computer Engineering Department, BINUS ASO School of Engineering, Bina Nusantara University, Tangerang, Banten 15325 Indonesia; 6 Fukui Science Education Academy, Takagi Chuou 2 choume, Fukui, 910-0804 Japan; 7 Research Center of Maju Makmur Mandiri Foundation, 40/80 Srengseng Raya, Jakarta, 11630 Indonesia

**Keywords:** Zircaloy-4 tube, Non-destructive H analysis, Electric field, He metastable excited states, LIBS, Optical spectroscopy, Laser-produced plasmas

## Abstract

We conduct an experimental study to search for the urgently needed method for routine, no sample pretreatment, in-situ, and less-destructive analysis of Hydrogen (H) content in Zircaloy-4 tube used as radioactive fuel container in light water nuclear power plant. For this purpose, we implemented laser-induced breakdown spectroscopy (LIBS) in Helium (He) surrounding gas at a relatively low pressure of around 3 kPa and low laser energy of 17 mJ. In addition, we designed a new compact and portable sample chamber accommodating an open end so the chamber can tightly fit the Zircaloy-4 tube surface. Inside the chamber we put electrodes to apply an additional electric field. We found that applying an electric field in the plasma expansion region increases the H emission intensity by a factor of 6. Consequently, the H I 656.2 nm emission line obtained from the Zircaloy-4 sample containing H of 11 ppm impurity featuring a sharp linewidth (0.1 nm) with high signal-to-noise ratio (S/*N* = 120). Thus, it offers potential applications to non-destructive H analysis in Zircaloy-4 tubes used in light water nuclear power plant. The detection limit of H was estimated less than 0.26 ppm, well below the sensitivity limit of around 600 ppm required for the regular inspection of Zircaloy-4 tubes in light water nuclear power plant.

## Introduction

Nuclear energy offers distinct advantages over other emerging energy sources, making it a crucial solution for meeting the growing demand for large-scale, high-efficiency energy production. Although rare and isolated nuclear accidents have occurred in the past, the construction and development of new nuclear power plants continue. These modern facilities are equipped with significantly enhanced safety systems, with many utilizing light water moderators to improve cost-effectiveness. Despite these advancements, optimizing reactor efficiency remains a persistent challenge. In nuclear reactors, uranium fuel is housed within Zircaloy-4 tubes submerged in water. During operation, the interaction between the hot water and the Zircaloy-4 surface leads to the formation of zirconium oxide and hydrogen gas, which permeates and accumulates within the tubes. Excessive hydrogen buildup weakens the structural integrity of the Zircaloy-4 tubes, reducing their mechanical strength and potentially compromising reactor safety^1–5^. As a result, regular monitoring of hydrogen concentrations within Zircaloy-4 tubes is essential to ensure the safe and efficient operation of nuclear reactors^6–8^.

The conventional method for detecting hydrogen (H) involves the use of a gas detector, which requires melting a section of the Zircaloy-4 tube in a carbon furnace and handling the residual fuel. This process is labour-intensive, time-consuming, and destructive. Consequently, there is a strong need for a more efficient and less invasive alternative to improve reactor maintenance.

Recent advancements have shown that hydrogen detection can be enhanced through modifications to laser-induced breakdown spectroscopy (LIBS)^9–19^ a widely used tool for rapid spectrochemical analysis in both industrial and research settings^20–23^. By utilizing atmospheric pressure helium (He) gas and a double-pulse configuration, the clear detection of the H I 656.2 nm emission line from Zircaloy samples was successfully achieved^14,19,24^. This development addressed long-standing challenges, such as Stark broadening and reduced emission intensity caused by the time mismatch effect commonly encountered in conventional LIBS conducted in atmospheric air^25^. The time mismatch effect occurs when fast-moving atoms ablated from the target pass through the plasma before they can participate in the shock wave-induced thermal excitation necessary for enhanced emission, a particular issue for light, fast-moving atoms like hydrogen. By leveraging metastable He excited states in high-pressure He plasma, these challenges were overcome, enabling the delayed and prolonged detection of hydrogen’s atomic emission^12,14,24,26,27^. This approach relies on a He-assisted excitation (HAE) process, where the emission enhancement is facilitated through a Penning-like energy transfer mechanism in cooled He plasma, as supported by recent studies demonstrating HAE-induced emission improvements in other elements^14,24,26,28^. Such Penning-like mechanisms are widely used in gas discharge lamps and lasers, gas electron multipliers, and radiation detectors^29–33^.

Although the promising results in previous studies were achieved using an orthogonal double-pulse configuration in an atmospheric helium (He) environment, this setup requires either two lasers or a single laser with an additional optical system to split the beam into two orthogonal paths of significantly different lengths^34,35^accomplished through a complex arrangement of mirrors. Such technical demands render this approach impractical for in-situ inspection of Zircaloy tubes, limiting its viability as a suitable alternative to current methods.

Previous experiments have addressed this limitation by eliminating the need for a double-pulse configuration and a high-pressure environment through the use of low-pressure He gas^27,36–40^. However, the use of a bulky sample chamber in these studies remains a significant obstacle, making the method unsuitable for in-situ hydrogen detection in Zircaloy tubes. Consequently, further improvements to the experimental method are necessary to overcome these technical challenges and support the efficient operation and maintenance of light water nuclear reactors.

This study used a modified compact chamber to address these issues. The chamber was designed to tightly fit the sample surface against its open end and was equipped with electrodes to generate an external electric field around the target plasma. As has been reported by some researchers, introducing the electric field in the plasma can enhance the emission intensity of some elements in the plasma^41–44^. In our case, we utilized the electric field to accelerate the charged particles within the plasma to increase the excited H atoms and the number of metastable He excited state atoms through collisions. This proposed method simplified the complexity of the previous double-pulse configuration. The collision between the charged particles and H atoms, along with the Penning-like energy transfer mechanism between metastable He atoms and H atoms in the plasma, enhanced the number of excited H atoms. As a result, the H I 656.2 nm emission intensity increased by a factor of six.

## Experimental methods

**Fig. 1:**
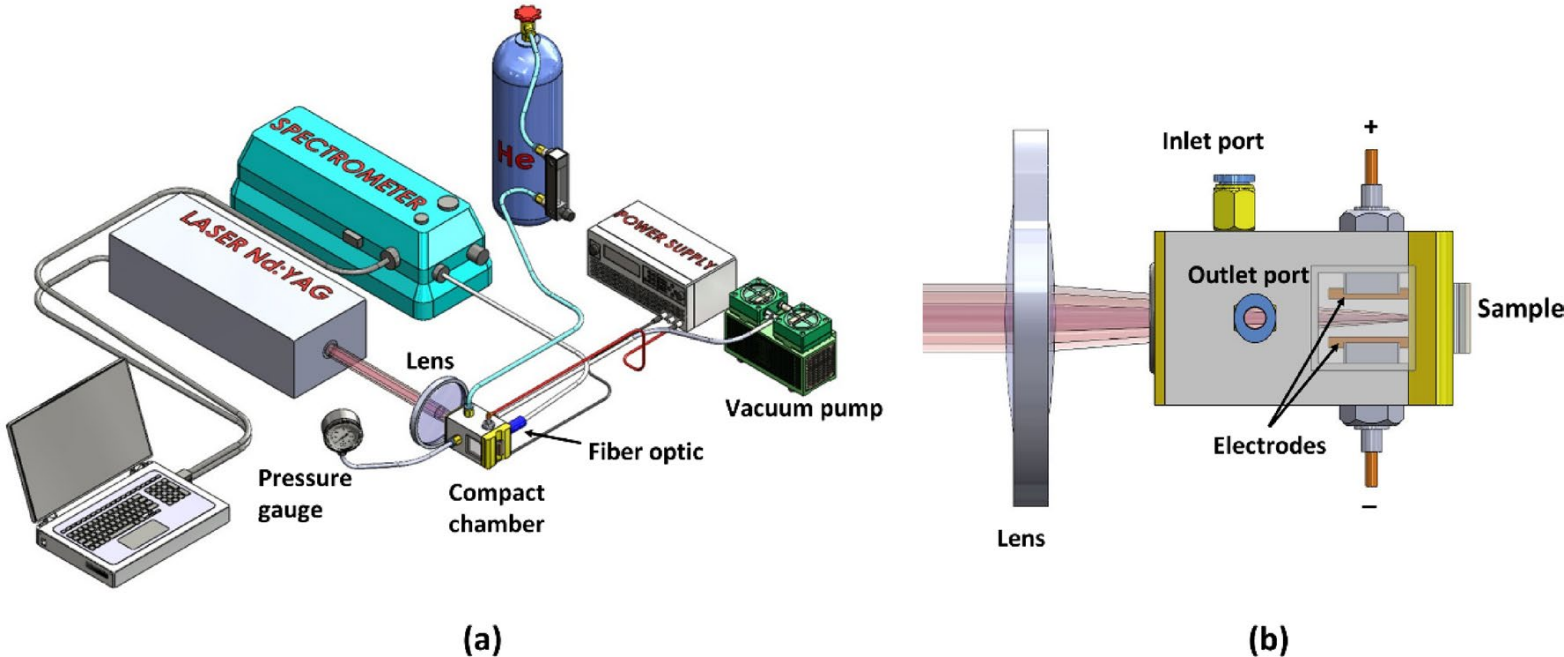
(**a**) Schematic of experimental configuration and (**b**) schematic design of a compact chamber.

Figure 1 shows the schematic diagram of the experimental arrangement used in the present work. In this experiment, the ns 355 nm Nd: YAG laser (Quanta Ray, INDI-10, 5 ns, maximum energy of 100 mJ) was operated with its output energy fixed at 17 mJ. The target plasma was created by focusing the laser beam using a lens with a 150 mm focal length. For in-situ application in the light-water nuclear power reactor, we specially designed a compact sample chamber made of brass with dimensions of 80 mm (length) × 50 mm (width) × 50 mm (height).

The chamber had a quartz window on one end for the passage of the laser pulses and a concave open end on the other side, which was equipped with an O-ring that could be tightly fitted to the surface of the Zircaloy-4 tube. Additionally, the chamber had gas outlet and inlet ports to facilitate evacuation and allow the continuous flow of helium gas in the chamber at a certain flow rate. A mini vacuum pump (Ulvac, model MiniDia pump) was employed to evacuate the chamber, and a digital absolute vacuum meter was used to monitor the He pressure inside the chamber. The chamber was also equipped with electrodes composed of two rectangular copper plates separated by 10 mm. The electrodes were connected to the high-voltage DC source to generate an electric field between the plates. Before flowing the high-purity helium gas, the chamber was heated to 473 K for 30 min to eliminate the H contribution from surface water inside the chamber.

The emission spectrum from the plasma was captured using a 100 μm diameter optical fiber positioned at a fixed distance of 7 cm laterally from the plasma. The other end of the fiber was connected to a detection system comprising a spectrograph (McPherson, model 2061, f = 100 cm, Czerny-Turner configuration) and an Optical Multichannel Analyzer (OMA, Andor ICCD with a 256 × 1024 pixel array) for emission line detection. The OMA operated with a 30 µs gate width and a 1 µs gate delay. To minimize the impact of the laser energy fluctuation on spectral intensities, the emission spectrum is averaged over 200 laser shots. To analyze the dynamic behavior of the emission intensity, the spectrograph’s output mirror was redirected to a photomultiplier tube (PMT, Hamamatsu IP 28, 2 ns rise time). For this purpose, the spectrograph’s center wavelength was adjusted to the target wavelength, with the entrance slit set to 40 μm and the exit slit to 40 μm for the PMT, while the PMT high voltage was maintained at 600 V. A fast preamplifier (SRS model 240) with a 125× gain was used to amplify the PMT output signal. A digital oscilloscope was used to display the electronic signal output from the fast amplifier. To reduce the influence of the laser energy fluctuation, the time-resolved data is obtained by averaging 16 acquisitions on the oscilloscope.

A porous alumina cylinder with a diameter of 1 cm was used as the sample to study the emission characteristics of H under the proposed method. Finally, we used a Zircaloy-4 tube of 10 mm length, and 10 mm outer diameter, doped with 11 ppm of H (as measured using gas chromatography), as a sample to simulate the potential application of the proposed technique for H analysis in Zircaloy-4 tubes in light water nuclear power plant.

## Results and discussion

**Fig. 2:**
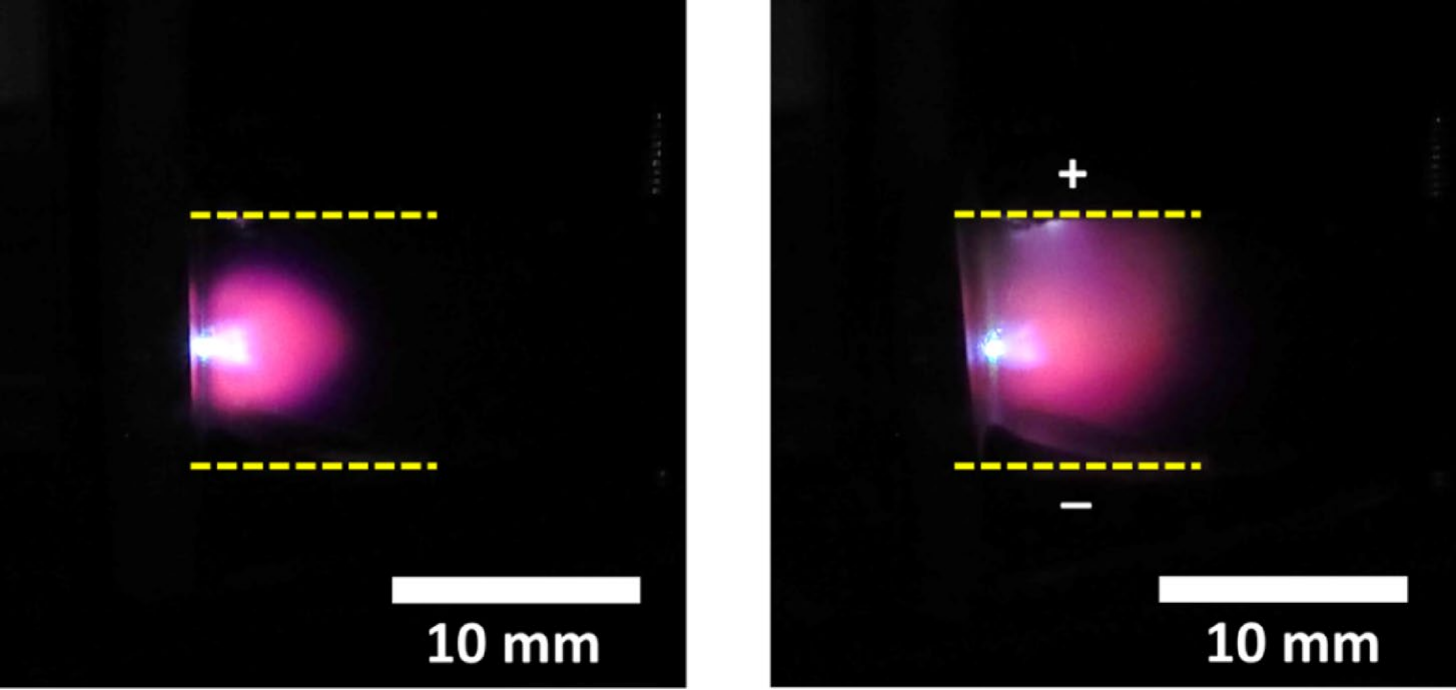
Photograph of the porous alumina plasma produced in the absence (left) and in the presence (right) of an external electric field.

Figure 2 shows the plasma photograph of a porous alumina sample both without and with an electric field in a 3 kPa He gas environment. The plasma volume inside the electrodes significantly increased when the electric field was applied by connecting the electrodes with a 300 V DC source. The pink color seen in the plasma is attributed to the combination of red emission from H atom (H I 656.2 nm) and orange emission from helium (He I 587.6 nm).

**Fig. 3:**
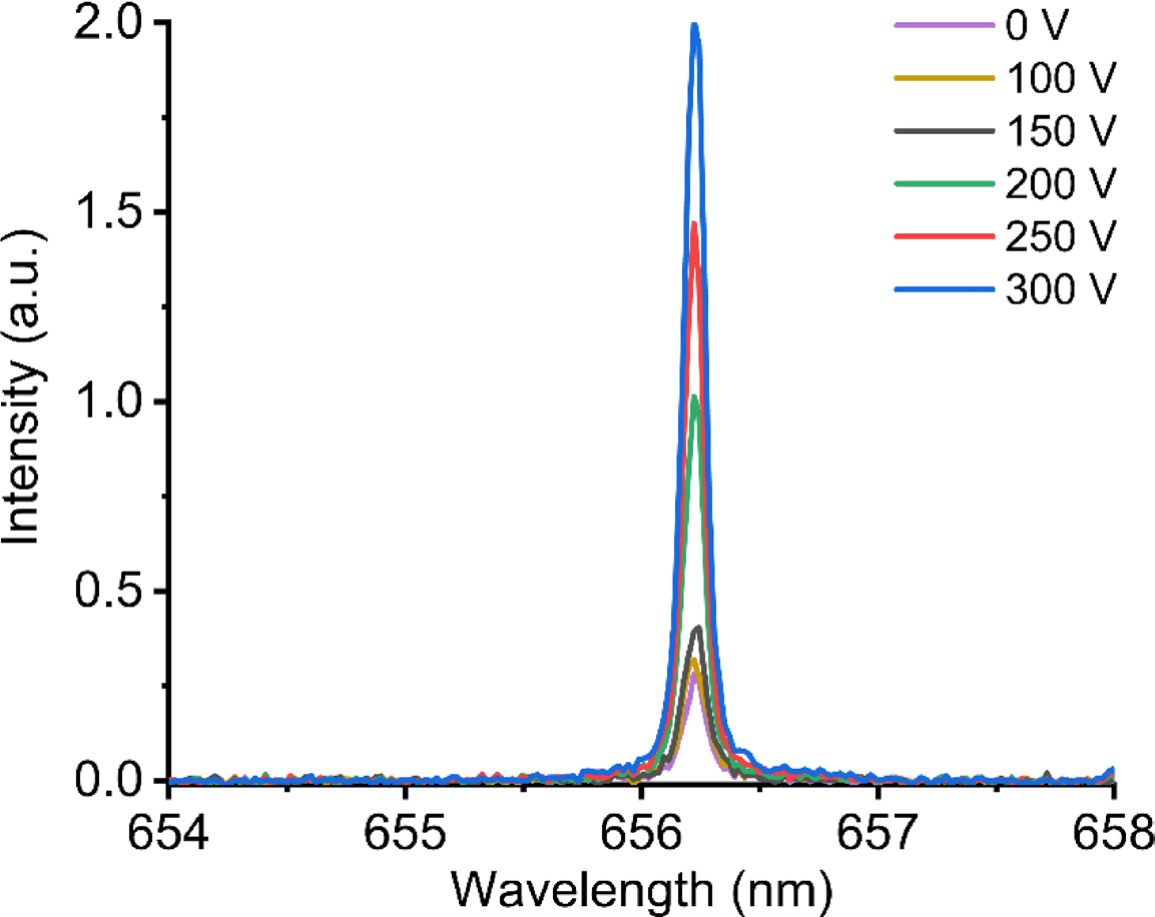
Emission spectra of H I 656.2 nm with varying applied voltage between two electrodes from 100 V to 300 V.

To observe the effect of electric field strength on the H I 656.2 nm emission intensity, we varied the applied voltage across the electrodes. We assume that the electric field strength between two electrodes is proportional to their applied voltage difference. Figure 3 shows the emission spectra of H I 656.2 nm when we vary the applied voltage between two electrodes from 100 V to 300 V. Under our experimental condition, we found that a significant increase of H I 656.2 nm emission was observed when the applied voltage on the electrodes was set above 200 V. The emission intensity of H keeps increasing until the applied voltage reaches 300 V. Beyond 300 V, the current follower starts to appear, making the plasma emission unstable. Therefore, in the following experiment, we used 300 V as the applied voltage on the electrodes.

**Fig. 4:**
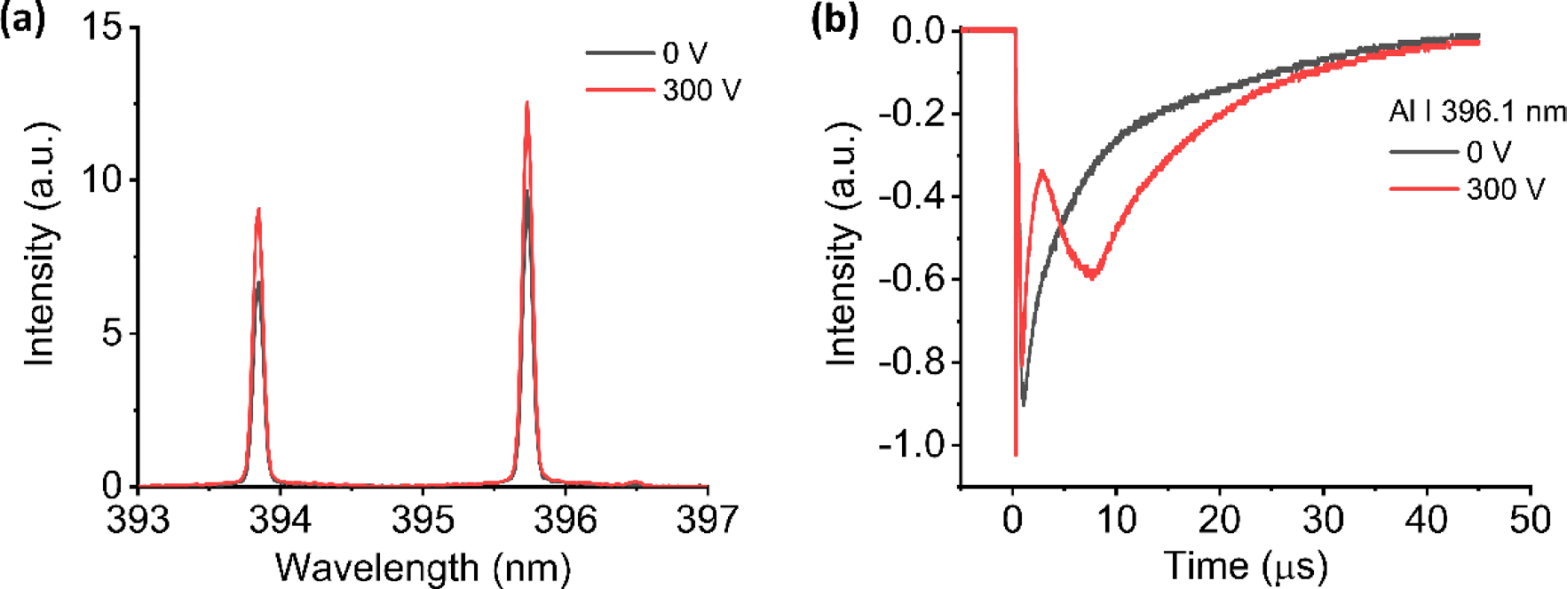
(**a**) Emission spectrum of Al I 394.4 nm and Al I 396.1 nm and (**b**) temporal profile of Al I 396.1 nm from porous alumina sample without and with the presence of electric field in He ambient gas.

To study the electric field’s role in enhancing H I 656.2 nm emission intensity, we investigate the spectral emission together with the dynamical behavior of Al I 396.1 nm as the host element, He I 587.6 nm as the ambient gas element, and H I 656.2 nm as the impurity element. Figures 4(a) and 4(b) show the spectral emission and dynamical behavior of Al I 396.1 nm, respectively. The emission intensity of Al I 396.1 nm increased 1.3 times when the electric field is applied between two electrodes. By looking at the time profile of Al I 396.1 nm, we noticed that, without the applied electric field, the emission intensity rises in a short time and then declines at a slow rate follow the typical excitation and cooling stages in the laser-induced shock wave plasma in low pressure He gas environment. On the other hand, when the electric field is applied, different behavior is observed, where the intensity drops very rapidly during the early times and then rises again before finally decreasing at a slow rate.

We speculate that the external electric field accelerates the charged particle in the plasma towards the electrodes and increases the probability of collision between charged particles in the plasma. When the sample plasma enters the electrode region, the collision between charged particles will increase the number of aluminum ions and reduce the number of neutral aluminum atoms. Consequently, the emission intensity of neutral aluminum atoms drops rapidly. The increased collisions between charge particles in the electrode region also increase the number of metastable excited state He atoms, which have a long lifetime. When the plasma cools down, the aluminum ion will transition to the ground state. The interaction between the aluminum ground state atoms and metastable excited He atoms will boost the number of neutral excited state Al atoms through Penning-like energy transfer process. This results in a secondary rise in the emission intensity of neutral aluminum atoms, which then declines again at a slow rate. This phenomenon is expected to contribute to a slight enhancement in Al I 396.1 nm emission.

**Fig. 5:**
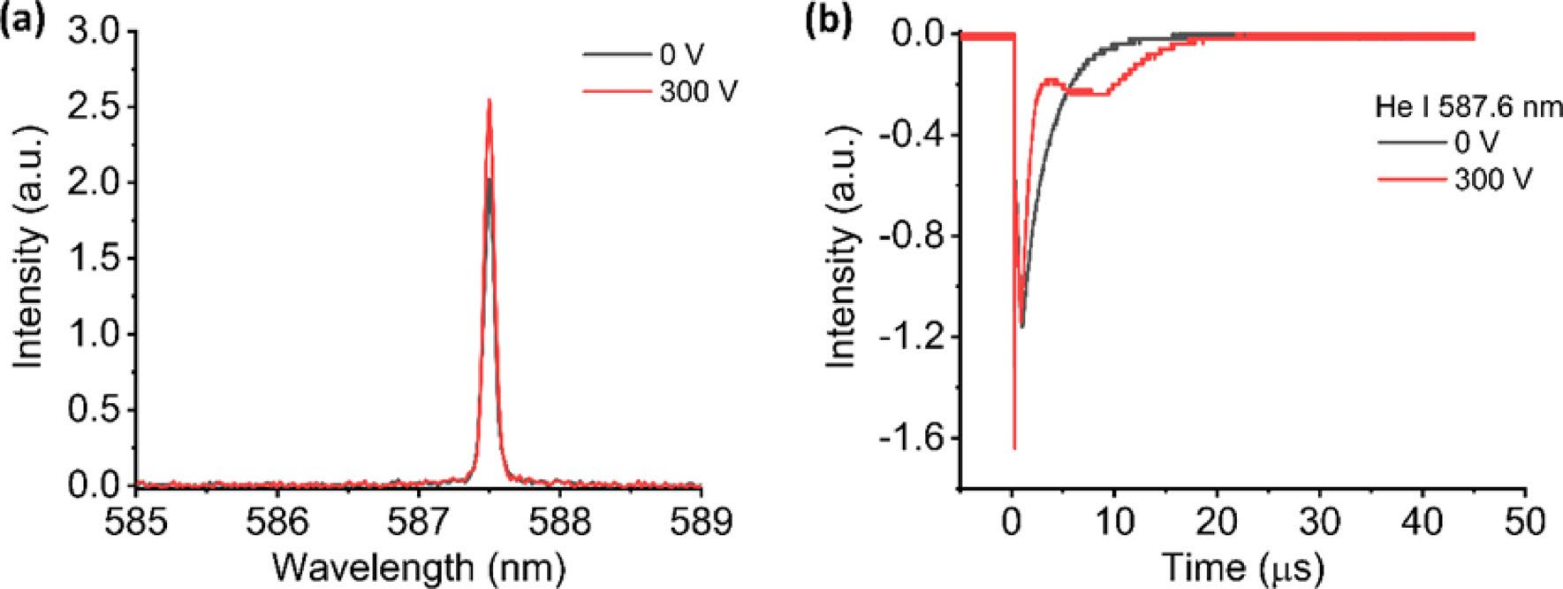
(**a**) Emission spectrum and (**b**) temporal evolution of He I 587.6 nm without and with the presence of electric field.

We also observed similar behavior for the emission spectrum and time profile of He I 587.6 nm, depicted in Fig. 5 (a) and (b). The emission intensity of He I 587.6 nm increased 1.3 times when the electric field is applied between two electrodes. When the external electric field was applied, it is expected that the collision between two metastable excited states He atoms increases the number of He ions and reduces the number of higher excited states He atoms. This resulted in the rapid decay of He I 587.6 nm emission. The He ions then can recombine with free electrons to produce higher energy state of He atoms. Through cascade transition, this higher energy He atoms can produce the metastable excited states He atoms. Consequently, the secondary rise of He I 587.6 nm emission was observed which contributed to the slight enhancement of He I 587.6 nm emission.

**Fig. 6:**
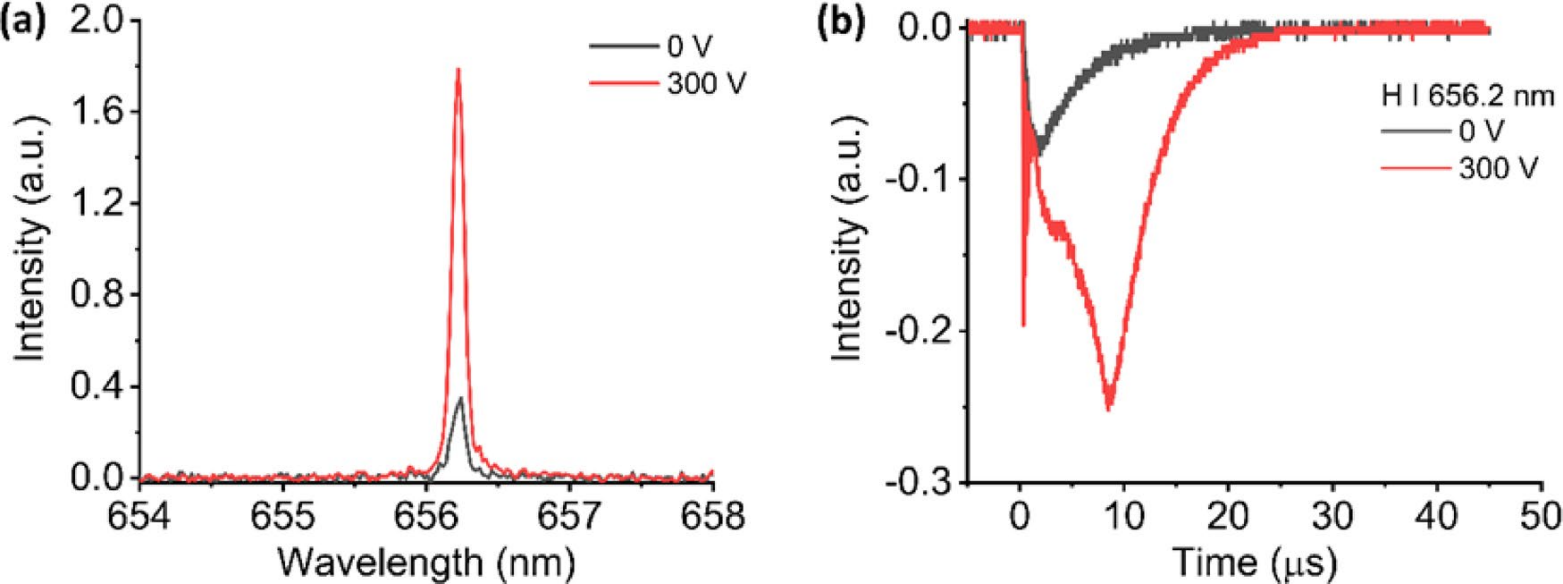
(**a**) Emission spectrum and (**b**) temporal evolution of H I 656.2 nm from porous alumina sample without and with the presence of electric field in He ambient gas.

However, we observed different behavior for H I 656.2 nm atoms, as shown in Fig. 6(a) and (b). A six (6) times emission enhancement of H I 656.2 nm was observed when the external electric field was applied to the target plasma. Since H atom has a large ionization energy compared to aluminum, it has a low probability of becoming ionized through collision with charged particles in the electrode region. Consequently, there is no rapid drop observed in the temporal profile of H I 656.2 nm. In this case, collision with charge particles only increases the number of excited states H atoms. At later stage, the interaction between the ground state H atoms with the metastable excited states He atoms through Penning-like energy transfer further increase the H I 656.2 nm emission intensity as demonstrated in its time profile. The higher enhancement observed in H I 656.2 nm compared to Al I 396.1 nm is attributed to the higher probability of the Penning-like energy transfer in H atoms compare to the Al atoms.

**Fig. 7:**
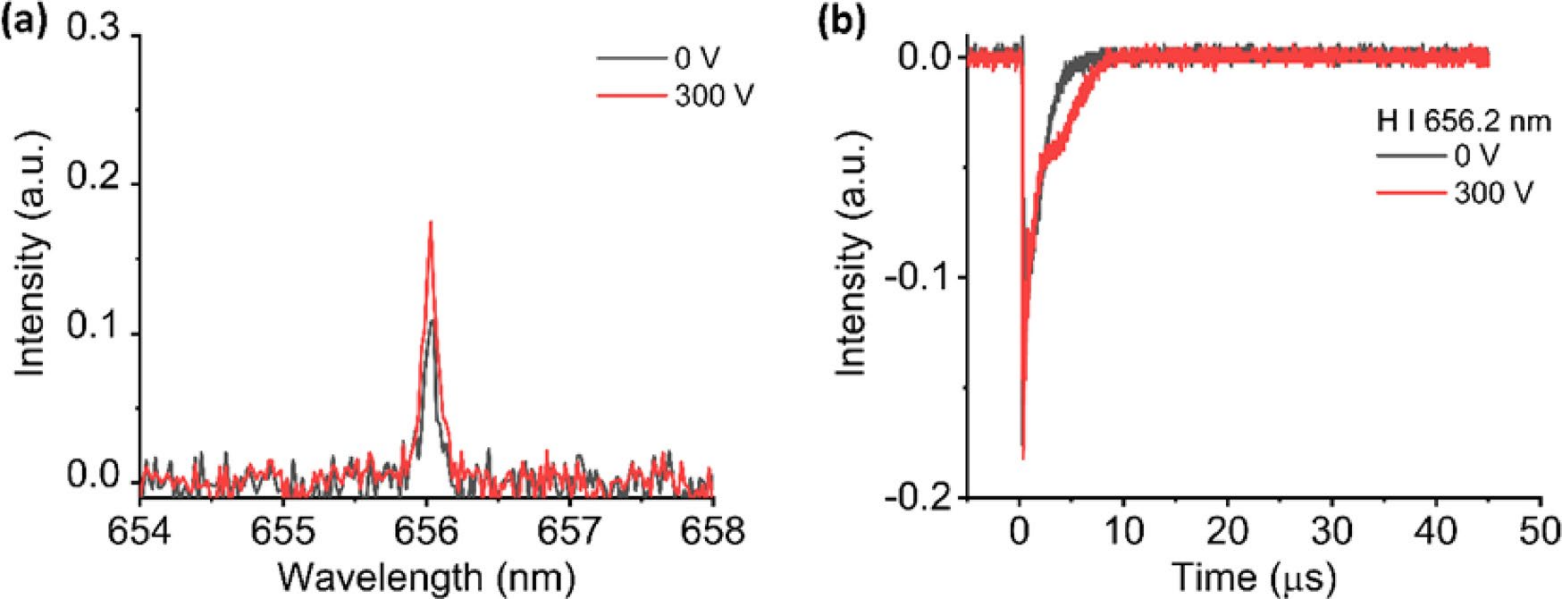
(**a**) Emission spectrum and (**b**) temporal evolution of H I 656.2 nm from porous alumina sample without and with the presence of electric field in N_2_ ambient gas.

To prove that the metastable excited states of He atoms play an essential role in enhancing the H I 656.2 nm emission intensity, we conducted a similar experiment by replacing the He with N_2_ as the surrounding gas. Figures 7 (a) and (b) show the emission spectrum and the H I 656.2 nm temporal evolution without and with the applied electric field. Compared with the He environment, the H I 656.2 nm emission intensity is lower in the N_2_ environment. Besides, the emission intensity of H I 656.2 nm only enhanced 1.6 times when the electric field was present between the electrodes, highlighting the diminished enhancement effect in the absence of metastable He states. This result underscores the importance of metastable excited-state He atoms in facilitating the observed emission enhancement.

**Fig. 8:**
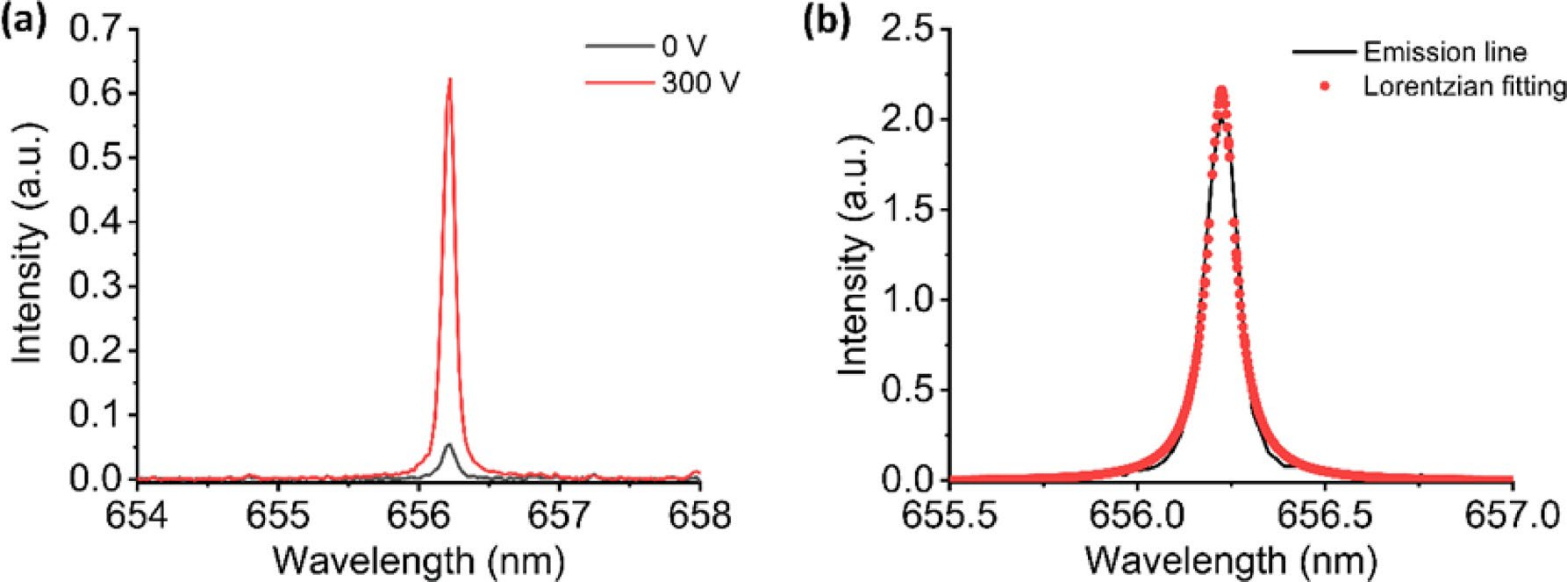
(**a**) Emission spectrum of H I 656.2 nm from Zircaloy-4 sample without and with the presence of electric field in He ambient gas and (**b**) The Lorentzian fit of the emission spectrum.

Finally, we used a Zircaloy-4 tube containing 11 ppm of H as the sample. The emission spectra of H I 656.2 nm without and with the applied electric field are presented in Fig. 8 (a). The enhancement of H I 656.2 nm emission intensity was observed around 10 times in this case. This enhancement can be attributed to the hardness of the Zircaloy-4 material, which is greater compared to that of porous alumina. By using the conventional definition of the limit of detection (LOD) as the ratio of signals to three times baseline noise, we estimated the LOD from the emission spectrum to be 0.26 ppm. Additionally, from the emission spectrum, we calculated the signal-to-noise ratio of 120 and linewidth of 0.1 nm as shown in Fig. 8 (b).

## Conclusion

A compact and portable chamber equipped with electrodes was developed for in situ analysis of H in Zircaloy-4 tube in light water nuclear power plant. We have demonstrated the application of an external electric field to enhance the emission intensity of H in low pressure He gas. The presence of an electric field increased the number of excited states H atoms and enhanced the production of metastable excited states He atoms through collision. The interaction between H and metastable excited state He atoms contribute to the H I 656.2 nm emission enhancement. In summary, the proposed method shows potential for in situ analysis of H in Zircaloy-4 tubes used in light water nuclear power plants. Further long-term testing will be essential to validate its applicability in operational environments.

## Data Availability

All data generated or analyzed in this study are contained within this published article.
